# Umbilical Cord-derived Mesenchymal Stem Cells Instruct Monocytes Towards an IL10-producing Phenotype by Secreting IL6 and HGF

**DOI:** 10.1038/srep37566

**Published:** 2016-12-05

**Authors:** Yinan Deng, Yingcai Zhang, Linsen Ye, Tong Zhang, Jintao Cheng, Guihua Chen, Qi Zhang, Yang Yang

**Affiliations:** 1 Department of Hepatic Surgery, The Third Affiliated Hospital of Sun Yat-sen University, Guangzhou, China; 2 Guangdong Provincial Key Laboratory of Liver Disease Research, Guangzhou, China; 3 Cell-gene Therapy Translational Medicine Research Center, The Third Affiliated Hospital of Sun Yat-sen University, Guangzhou, China

## Abstract

Human UC-MSCs are regarded as an attractive alternative to BM-MSCs for clinical applications due to their easy preparation, higher proliferation and lower immunogenicity. However, the mechanisms underlying immune suppression by UC-MSCs are still unclear. We studied the mechanism of inhibition by UC-MSCs during the differentiation of monocytes into DCs and focused on the specific source and the role of the involved cytokines. We found that UC-MSCs suppressed monocyte differentiation into DCs and instructed monocytes towards other cell types, with clear decreases in the expression of co-stimulatory molecules, in the secretion of inflammatory factors and in allostimulatory capacity. IL6, HGF and IL10 might be involved in this process because they were detected at higher levels in a coculture system. UC-MSCs produce IL-6 and HGF, and neutralization of IL-6 and HGF reversed the suppressive effect of UC-MSCs. IL10 was not produced by UC-MSCs but was exclusively produced by monocytes after exposure to UC-MSCs, IL-6 or HGF. In summary, we found that the UC-MSC-mediated inhibitory effect was dependent on IL6 and HGF secreted by UC-MSCs and that this effect induced monocyte-derived cells to produce IL10, which might indirectly strengthen the suppressive effect of UC-MSCs.

Mesenchymal stem cells (MSCs) are were first identified in bone marrow. Bone marrow-derived MSCs (BM-MSCs) are capable of differentiation into bone, cartilage and other mesenchymal tissues^1^. Importantly, they display striking immunological characteristics, including low immunogenicity and immunoregulatory properties^2^,^3^,^4^, that have been the subject of many studies over the past decade, making BM-MSCs ideal candidates for treating immunological diseases.

It has been widely shown that BM-MSCs suppress NK-cell proliferation and cytotoxicity^5^ and impair T cell activation and proliferation^6^,^7^,^8^. Soluble factors proposed to be involved in this effect include indoleamine 2, 3-dioxygenase, prostaglandin E2, TGF-β1, IL-6 and nitric oxide^9^,^10^,^11^.

However, aspiration of bone marrow is difficult and involves invasive procedures, which restrict the application of BM-MSCs. Thus, there is growing interest in finding alternative sources of MSCs. Human umbilical cord (UC) contains multi-potent stromal cells also known as UC-MSCs^12^. Compared with MSCs isolated from bone marrow, UC-MSCs offer distinct advantages, including easier accessibility, more primitive properties, higher proliferation capacity and lower immunogenicity^13^. Due to these advantages, UC-MSCs are being explored as a promising candidate for many potential clinical applications. Recent studies have provided encouraging results regarding the utility of UC-MSCs in several disease models, such as rescuing visual functions in a rodent model of retinal disease^14^, alleviating neuropathic pain^15^, protect against experimental colitis^16^ and treating rat liver fibrosis^17^,^18^. The use of UC-MSCs as a cellular therapy is currently being explored in clinical trials, including for the treatment of GvHD^19^,^20^.

Recent studies have shown that UC-MSCs, such as BM-MSCs, can suppress T cell activation and proliferation though a PGE2-dependent manner^21^. However, the immune suppression effect of UC-MSCs on DC differentiation is still poorly understood. Our early studies found that UC-MSCs induced DCs to differentiate into tolerogenic DCs through the upregulation of SOCS1 and that IL6 in coculture supernatant was involved in the UC-MSC immunoregulatory effect on DC transdifferentiation^22^. However, neither we nor other researchers have identified the cells that produce these cytokines. Therefore, the specific role of these cytokines in MSC-mediated immune suppression is still unclear.

In the current study, we found that UC-MSCs suppressed monocyte differentiation into DCs and instructed monocytes towards IL10-producing cell types, with a clear decrease in the expression of co-stimulatory molecules, in the secretion of inflammatory factors and in allostimulatory capacity. Furthermore, IL6, HGF and IL10 might be involved in this process because they were detected at higher levels in coculture. UC-MSCs produced IL-6 and HGF, and IL-6 and HGF neutralization reversed the suppressive effect of UC-MSCs. IL10 was not produced by UC-MSCs, but was exclusively produced by monocytes after exposure to UC-MSCs, IL-6 or HGF. In summary, we found that the UC-MSC-mediated inhibitory effect was dependent on IL6 and HGF and that this effect subsequently induced monocyte-derived cells to produce IL10, which might indirectly strengthen the suppressive effect of UC-MSCs.

## Materials and Methods

### Culture of human umbilical cord-derived mesenchymal stem cells

Human UC-MSCs were isolated and identified as previously described^14^. Briefly, fresh human umbilical cords were obtained, cut into 0.5-cm pieces and floated in Dulbecco’s modified Eagle’s medium containing low glucose (Life Technologies, Carlsbad, CA), 10% fetal bovine serum (FBS; Life Technologies, Carlsbad, CA), 100 U/ml penicillin and streptomycin (P/S; Invitrogen Corp) at 37 °C in a humidified atmosphere with 5% CO_2_. The medium was changed every 2 d, and non-adherent cells were removed by washing after 7 d. When well-developed colonies of fibroblast-like cells appeared after 10 d, the cultures were trypsinized and transferred (without dilution) into a new flask for further expansion. When UC-MSCs reached confluency, the cells were detached, characterized by FACS analysis and used in the co-culture experiments at passage 2–5. The study was approved by the National Ethics Committee of the Third Affiliated Hospital of Sun Yat-sen University. The patients’ informed consent was obtained. All experiments were performed in accordance with relevant guidelines and regulations.

### Isolation of human CD14+ monocytes

Peripheral blood mononuclear cells (PBMCs) were isolated from healthy blood by Ficoll-Paque (1.077 g/mL; Invitrogen) density gradient centrifugation. Monocytes (more than 90% CD14+) were purified from PBMCs using the MACS Monocyte Isolation Kit (Miltenyi Biotec, Bergisch Gladbach, Germany). The study was approved by the National Ethics Committee of the Third Affiliated Hospital of Sun Yat-sen University. The patients’ informed consent was obtained.

### Differentiation of monocytes

Freshly isolated monocytes (CD14+) were cultured at a concentration of 4.0 × 10^5^ cells/well in 12-well plates containing 1 ml RPMI (Invitrogen Corp., Paisley, UK) with 100 U/ml penicillin and streptomycin (P/S; Invitrogen Corp), L-glutamine (Invitrogen Corp., Paisley, UK), 10% FBS and the growth factors IL4 (50 ng/ml, PeproTech, USA) and GM-CSF (50 ng/ml, PeproTech, USA) for 5 days, resulting in the generation of immature DCs (iDCs; CD14-/CD1a+). After the 5-day incubation, to gain mature DCs (mDCs), LPS (100 ng/ml, Sigma-Aldrich, USA) was added to stimulate the cells for 48 h.

To examine the effect of UC-MSCs on monocyte differentiation into mDCs, UC-MSCs treated with mitomycin were cultured at a concentration of 2.0 × 10^5^ cells/well in 12-well plates containing 1 ml RPMI for 24 h, and then monocytes, IL4 and GM-CSF were added with direct cell-cell contact or in a transwell system (pore size 0.4 μM; Corning Inc., Lowell, MA, USA) at a MSC-monocyte ratio of 1:10 for 5 days and then LPS was added to stimulate the cells for 48 h. After 7-day incubation, monocytes were the separated from the UC-MSCs by spinning the cells in suspension and then washing them.

To examine the effect of some soluble factors, IL10 (50 ng/ml), IL6 (50 ng/ml), HGF (50 ng/ml), anti-IL10 (5 μg/ml), anti-HGF or anti-IL6 (5 μg/ml) (R&D Systems Europe Ltd., Abingdon, UK) was added every 3 days.

### Flow cytometry

The antibodies used for flow cytometry were FITC-, PE- or APC-conjugated mouse anti-human CD3, CD4, HLA-DR, CD1a, CD14, CD80, CD86, and CD83, all of which were purchased from BioLegend. The cultured cells were collected, washed twice, and resuspended in 100 μl of PBS containing 0.1% BSA. The cells were then stained and labeled with either specific antibodies or the appropriate isotype controls, subsequently incubated on ice for 30 min, washed with PBS containing 0.1% NaN_3_ and 0.5% BSA, and then fixed with 1% paraformaldehyde solution. Analyses were performed using a FACScan and the CellQuest software (BD Bioscience). For the intracellular staining of T cells, peripheral blood lymphocytes (PBLs) were stimulated for 5 h with phorbol 12-myristate 13-acetate (PMA) plus ionomycin (Sigma-Aldrich, USA), and brefeldin A (10 mg/ml; Sigma-Aldrich, USA) was added during the last 3 h. The cells were washed and stained with monoclonal antibodies specific for CD4 for 20 min at 4 °C. After being washed with PBS, the cells were fixed and permeabilized with fixation/permeabilization solution (eBioSciences, San Diego, CA) for 15 min at room temperature. Following further washing, the cells were stained with monoclonal antibodies against IFN-γ, IL2 and TNFa (eBioSciences, San Diego, CA) for 20 min at room temperature.

### Cytokine assays

Coculture supernatants were collected on day 7, and the concentrations of IL6, IL10, and HGF in the supernatants were determined using ELISA kits (R&D Systems, Minneapolis, MN).

To determine the cytokine expression patterns of UC-MSC-treated monocytes, monocytes were collected at 7-day incubation and cultured at a concentration of 1.0 × 10^5^ cells/well in 12-well plates containing 1 ml RPMI for 24 h with or without LPS (100 g/ml) stimulation. Subsequently, the culture supernatants were collected, and the concentrations of IL17, IL10, TNFa and IL12p70 were determined using ELISA kits (R&D Systems, Minneapolis, MN).

### Mixed lymphocyte reaction

Once labeled with carboxyfluorescein diacetate succinimidyl diester (CFSE) (Invitrogen) and suspended in RPMI 1640 supplemented with 10% FBS, 100 U/ml penicillin, and 100 U/mL streptomycin, allogeneic PBLs from PBMCs were cultured at a concentration of 10^5^ cells per 200 μL per well in 96-well U-bottom plates containing 2 × 10^4^ monocytes treated with mitomycin. After 5 d, the cells were harvested and sorted by FACS.

### RT-PCR

Total RNA was extracted using TRIzol (Invitrogen) and then reverse transcribed using a QuantiTect RT Kit (Qiagen). The PCR products were analyzed by electrophoresis and ethidium bromide staining of a 2% agarose gel, which was subsequently photographed. In some experiments, gene expression was evaluated using a SYBR Green real-time PCR kit (Takara). Data were normalized to the reference gene β-actin.

gene forward reverse

IL6 5′-TTCAATGAGGAGACTTGCCTG-3′ 5′-ACAACAACAATCTGAGGTGCC-3′

IL10 5′-CCGAGATGCCTTCAGCAGAG-3′ 5′-GGTCTTGGTTCTCAGCTTGG-3′

HGF 5′-TACAGGGGCACTGTCAATACC-3′ 5′-CAGTAGCCAACTCGGATGTTT-3′

βactin 5′-AGGCATCCTCACCCTGAAGTA-3′ 5′- CACACGCAGCTCATTGTAGA-3′

### Statistical analysis

Statistical analyses were performed using Student’s t test. The data are depicted as the means ± SEMs, and p values ≤ 0.05 were considered significant. *p ≤ 0.05, **p ≤ 0.01, ***p ≤ 0.001. All statistical analyses were conducted using the Statistical Program for Social Sciences 13.0 software program (SPSS Inc., Chicago, IL).

## Results

### UC-MSCs instruct the differentiation of monocytes into CD14+CD1a− cells expressing low levels of co-stimulation molecules

**Figure 1:**
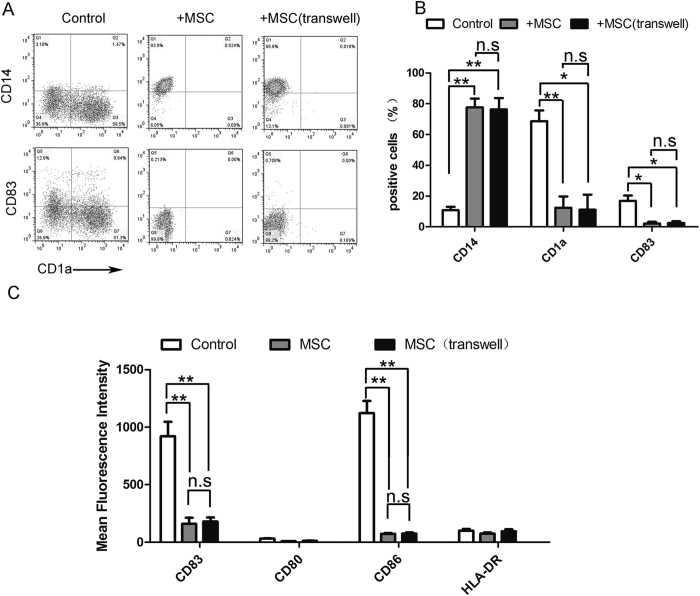
The effect of UC-MSCs on monocyte differentiation. After a 5-day incubation, monocytes were collected and analyzed by FACS. The CD14, CD1a, and CD83 expression in monocytes cultured alone (Control) or cocultured with UC-MSCs in direct cell-cell contact (+MSC) or in a transwell co-culture system (+MSC (transwell)) were tested (**A,B**). (**A**) One representative experiment of three is shown. (**B**) The data are shown as the means ± SEMs of three different healthy persons. After a 7-day incubation, monocytes were collected and analyzed by FACS, and the CD83, CD80, HLA-DR, and CD86 expression in monocytes cultured alone (Control) or cocultured with UC-MSCs in direct cell-cell contact (+MSC) or in a transwell co-culture system (+MSC (transwell)) were tested (**C**). The data are shown as the means ± SEMs of three different healthy persons.

Circulating CD14+ monocytes were cultured in the presence of GM-CSF and IL-4 with or without UC-MSC coculture. After 5 days of incubation, LPS was added for another 48 hours to promote the maturation of DCs. As expected, the monocytes differentiated into CD1a+CD14- iDCs (Fig. 1A,B) and further differentiated into fully mDCs after LPS stimulation (Fig. 1C). Following the addition of UC-MSCs to these cultures, the differentiation of monocytes towards DCs was inhibited. In the presence of UC-MSCs, monocytes retained high CD14 levels without acquiring CD1a and displayed lower levels of CD83 and of the costimulatory molecules CD80 and CD86 (Fig. 1).

### UC-MSCs alter cytokine production patterns of monocyte-derived cells

**Figure 2:**
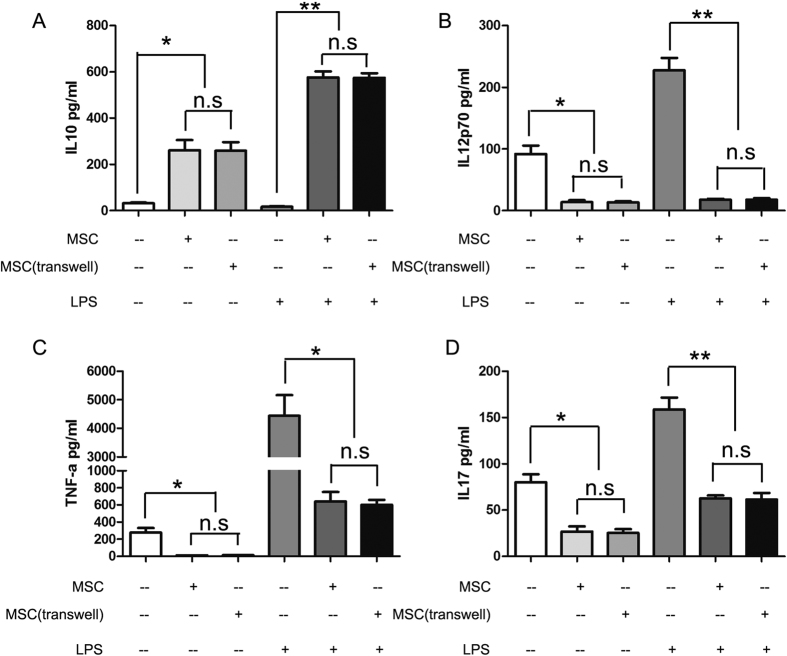
UC-MSCs alter cytokine production patterns of monocyte-derived cells. After a 7-day incubation, monocytes were collected and cultured with or without LPS for 24 h. The culture supernatants were then collected, and the concentrations of IL10 (**A**), IL12p70 (**B**), TNFa (**C**) and IL17 (**D**) were determined using ELISA kits. The data are shown as the means ± SEMs of three different healthy persons.

To further characterize the regulatory effect of UC-MSCs on monocytes, we determined the cytokine expression patterns of monocyte-derived cells by ELISA. After 7 days of incubation, monocytes were separated from UC-MSCs and culturing them with or without LPS for 24 hours; the culture supernatants were then collected for ELISAs. In contrast to monocyte-derived DCs, the monocyte-derived population generated in the presence of UC-MSCs showed higher IL10 expression and lower IL12p70, TNFa and IL17 expression (Fig. 2A,D).

### UC-MSCs impair the allostimulatory capacity of monocyte-derived cells

**Figure 3:**
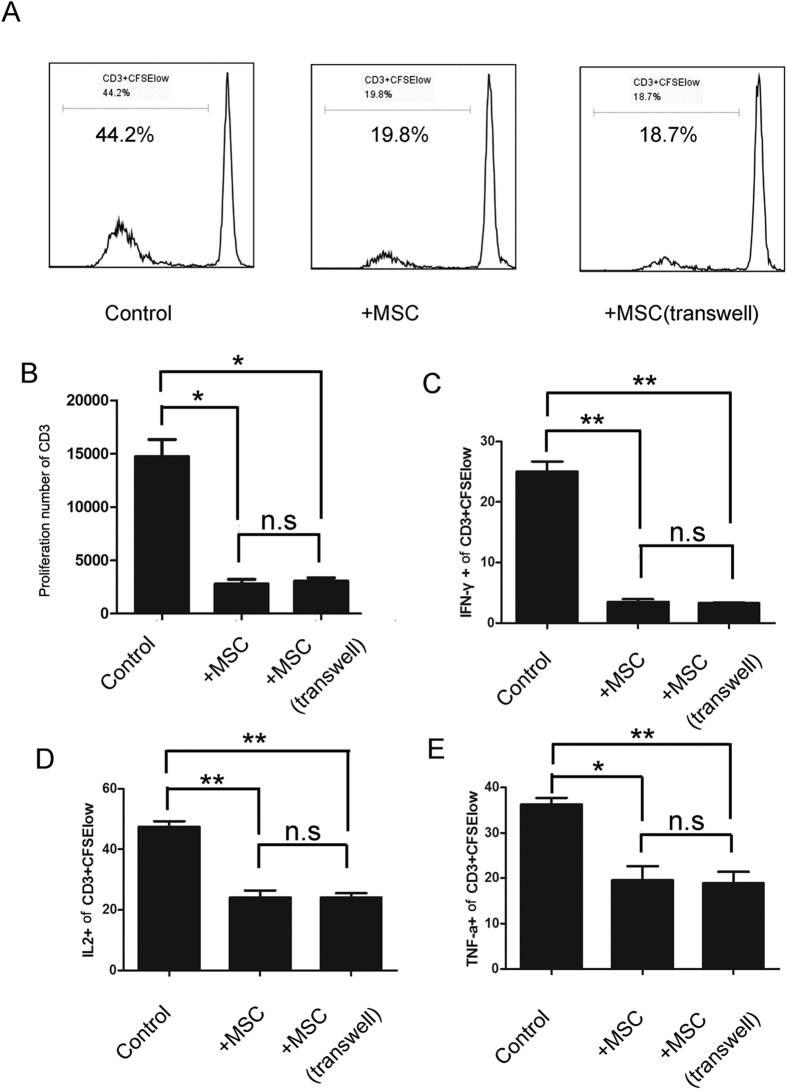
UC-MSCs impair the allostimulatory capacity of monocyte-derived cells. After a 7-day incubation, monocytes were collected and cultured with PBLs labeled with CFSE for 5 days. The cells were the collected, and the proliferation of CD3+ T cells was analyzed by FACS (**A,B**). (**A**) One representative experiment of three is shown. (**B**) The data are the proliferation numbers of CD3+ cells and the means ± SEMs of three different healthy persons. The ability of CD4+ T cells to produce IFNγ (**C**), IL2 (**D**), and TNFa (**E**) were tested by FACS. The data are shown as the means ± SEMs of three different healthy persons.

Next, the monocyte-derived populations in the absence or presence of UC-MSCs were functionally analyzed for their ability to stimulate the proliferation of allogeneic T cells. After 7 days of incubation, monocytes were separated from the UC-MSCs and cultured with allogenic PBLs labeled with CFSE for 5 days. As shown in Fig. 3, the monocyte-derived DCs effectively induced the proliferation of allogeneic CD3+ T cells (Fig. 3A,B). Furthermore, when in the presence of UC-MSCs, the monocyte-derived population exhibited a significantly decreased allostimulatory capacity compared to the monocyte-derived DCs (Fig. 3A,B). Moreover, our data showed that the expression of IFN-γ, IL2 and TNFa in CD4+CFSElow T cells was decreased significantly in the monocyte-derived populations in the presence of UC-MSCs when compared to the monocyte-derived DCs in the absence of UC-MSCs (Fig. 3C,E).

### UC-MSC-mediated inhibition of monocyte differentiation involves soluble factors

Studies have shown that MSC-mediated immunomodulation involves both cell contact-dependent and cell contact-independent mechanisms that are mediated through the release of soluble factors. To explore whether the immunoregulatory properties of UC-MSCs depend on direct contact with monocytes or if they are mediated by soluble factors, transwell experiments that prevented direct contact between purified CD14+ monocytes and UC-MSCs were performed. Our data showed that the transwell hardly prevented the suppressive effect of UC-MSCs on monocyte differentiation and function (Figs 1, 2, 3), indicating that UC-MSCs might exert their inhibitory effects mainly through soluble factors in the culture supernatant.

### The levels of IL6, IL10 and HGF are upregulated in co-cultures of monocytes and UC-MSCs

**Figure 4:**
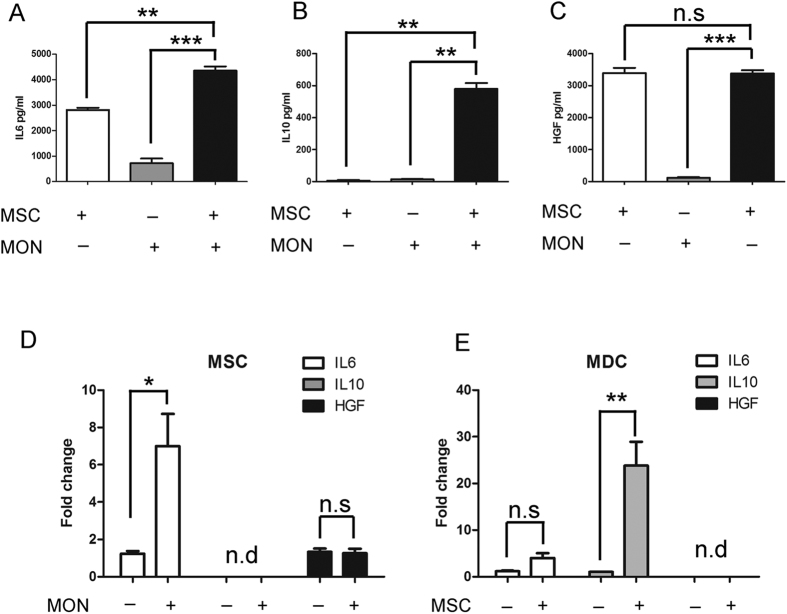
The source of IL6, IL10 and HGF in co-cultures was identified. After a 7-day incubation, coculture supernatants were collected, and the concentrations of IL6, IL10, and HGF in the supernatants were determined using ELISA kits (**A,C**). The data are shown as the means ± SEMs of three independent experiments. After a 7-day incubation, UC-MSCs (**D**) and monocytes (**E**) were collected to determine the mRNA levels of IL6, IL10, and HGF. “n.d” refer to “not detected”. The data are shown as the means ± SEMs of three independent experiments.

To identify the key factors responsible for the UC-MSC-mediated suppression of monocyte differentiation, the soluble factors present in the supernatants were analyzed. Monocytes cocultured with UC-MSCs in a transwell system had higher levels of HGF, IL6, and IL10 compared with monocytes alone (Fig. 4A,C).

To identify which cell population was responsible for the increased cytokine level, the mRNA expression of HGF, IL6 and IL10 in the UC-MSC and monocyte-derived cell populations was analyzed at day 7. Neither UC-MSCs nor UC-MSCs cultured in the presence of monocytes expressed IL10 mRNA (Fig. 4D). However, monocyte-derived DCs expressed IL10 mRNA, and this expression level was significantly upregulated after exposure to UC-MSCs (Fig. 4E). These data showed that the IL10 in the supernatants of MSC-monocyte co-cultures was secreted by the monocyte-derived cell population.

Next, we assessed the expression of IL6. Figure 4D shows that UC-MSCs express IL6 mRNA and that significantly increased IL6 mRNA expression is found in UC-MSCs after co-culturing with monocytes (Fig. 4D). Moreover, IL6 mRNA was observed in monocyte-derived DCs (Fig. 4E). However, the increase in IL6 expression in DCs cocultured with UC-MSCs was not statistically significant (Fig. 4E). These data indicate that the IL6 protein detected in the supernatants of monocyte-MSC co-cultures was produced both by the monocyte-derived cell population and by UC-MSCs.

Finally, we investigated the expression levels of HGF. As shown in Fig. 4D, the level of HGF mRNA was observed in UC-MSCs, but the expression was hardly increased when UC-MSCs were co-cultured with monocytes. Neither the monocyte-derived DCs nor the monocytes cultured in the presence of UC-MSCs expressed HGF mRNA (Fig. 4F). These data indicate that the HGF in the supernatants of monocyte-MSC co-cultures was produced by UC-MSCs.

### The role of HGF, IL6 and IL10 in the UC-MSC-mediated inhibition of monocyte differentiation

**Figure 5:**
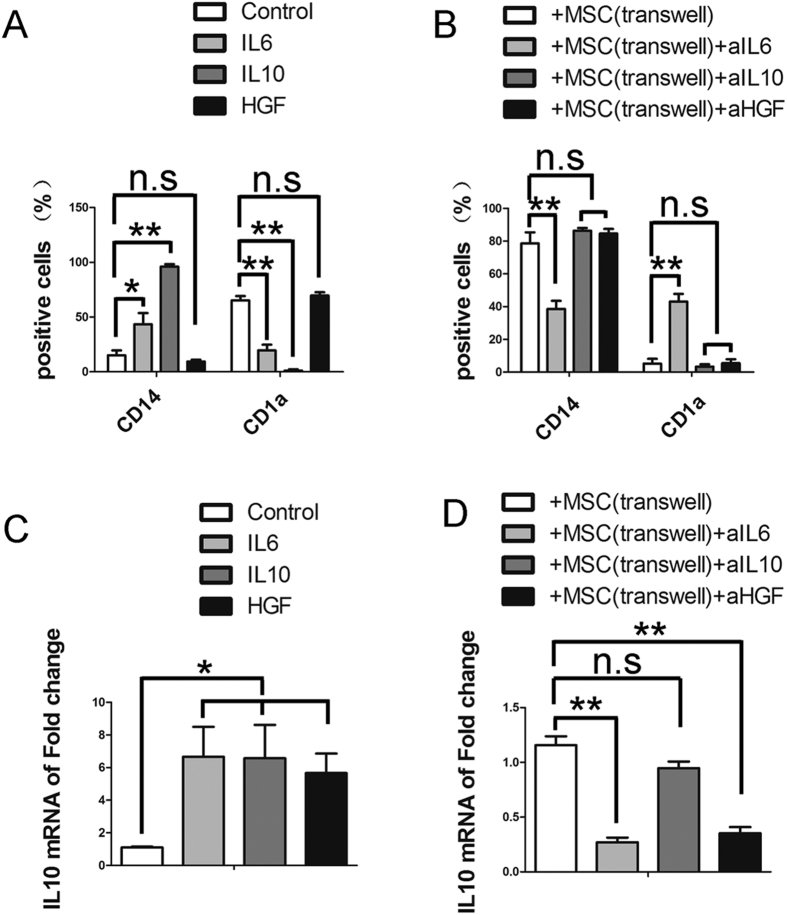
The role of HGF, IL6 and IL10 in the UC-MSC-mediated inhibition of monocyte differentiation. After a 5-day incubation, monocytes were collected and analyzed by FACS. The CD14 and CD1a expression in monocytes cultured alone (Control), with IL6, IL10, or HGF, or cocultured with UC-MSCs in a transwell co-culture system in the absence (+MSC (transwell)) or presence of anti-IL6, anti-IL10, or anti-HGF was determined (**A,B**). The data indicate the means ± SEMs of three independent experiments. After a 7-day incubation, monocytes cultured in the indicated conditions were collected to determine the mRNA levels of IL10 (**C,D**). The data are shown as the means ± SEMs of three independent experiments.

To determine the exact role of HGF, IL6 and IL10 in the inhibitory effect of UC-MSCs on monocyte differentiation, blocking antibodies against HGF, IL6 and IL10 were added to the transwell co-cultures. The addition of blocking antibodies against IL10 and HGF resulted in minor restoration of monocyte differentiation into CD14+CD1a− cells (Fig. 5B). However, the addition of blocking antibodies against IL6 resulted in a significant increase in CD1a expression and in a decrease in the expression of CD14 in the monocyte-derived population (Fig. 5B). The addition of both the HGF- and IL6-blocking antibodies substantially reduced the upregulation of IL10 mRNA expression in the monocyte-derived population (Fig. 5D).

We next investigated whether addition of HGF, IL6 or IL10 would inhibit monocyte differentiation. Both IL6 and IL10 significantly prevented monocyte differentiation into DCs, while HGF did not inhibit this process (Fig. 5A). Indeed, we found that addition of IL6 induced the expression of IL10 in monocytes (Fig. 5C), similar to the effect of UC-MSCs. Surprisingly, the addition of HGF significantly induced IL-10 expression in monocytes (Fig. 5C). These data show that MSC-derived IL6 is essential for the inhibition of monocyte differentiation by UC-MSCs and that both IL6 and HGF derived from UC-MSCs induce IL10 expression in monocytes.

## Discussion

Although BM-MSCs have outstanding immunoregulatory characteristics and thus have been used to treat immune diseases, their collection is difficult and involves invasive procedures. Thus, there is growing interest in finding alternative sources of MSCs and in exploring the therapeutic potential of these cells. UC-MSCs are of interest to scientists because they can be isolated and expanded easily in large quantities *in vitro*.

Currently, UC-MSCs are being explored as a promising candidate in clinical applications, e.g., in the treatment of GvHD. However, the mechanisms underlying immunoregulation by UC-MSCs are still unclear. In this study, we investigated the suppressive effect of UC-MSCs on monocyte differentiation into DCs and focused on the specific source and role of the involved cytokines.

Recent studies have shown that BM-MSCs might induce mature DCs to differentiate into regulatory DCs through the activation of the Notch pathway. Moreover, BM-MSCs might inhibit the differentiation and function of DCs^23^,^24^. In accordance with these findings, UC-MSCs suppressed monocyte differentiation into DCs in our system. The data derived from *in vitro* co-culture experiments of BM-MSCs and monocytes have shown that monocyte differentiation towards DCs can be inhibited by IL6^25^. Similarly, our data suggested that IL6 plays a role in the inhibitory effect of UC-MSCs on the differentiation of monocytes to DCs. To identify the source of IL6, we analyzed IL6 expression in monocytes and UC-MSCs and found that both are capable of IL6 production; therefore, the source of IL6 remained unclear. Because the suppressive effect of UC-MSCs, e.g., IL10 production and CD1a expression, could be reproduced by IL6, we considered UC-MSC-derived IL6 to be responsible for the MSC-induced inhibition. Indeed, the addition of IL6-blocking antibodies significantly reversed the suppressive effect of UC-MSCs. These data clearly indicated that UC-MSC-derived IL6 regulated monocyte differentiation *in vitro* and thereby instructed monocytes towards an IL10-producing cell type with decreased functional molecule expression and allostimulatory capacity. However, additional factors might play a role in the suppressive effect of UC-MSCs because the expression of functional molecules in monocyte-derived cells was not completely restored by IL6-blocking antibodies.

In our study, high concentrations of HGF secreted by UC-MSCs were detected in a coculture system. In murine allogeneic bone marrow transplantation, HGF ameliorated acute graft-versus-host disease (aGVHD) through the reduction of IL12 serum levels and suppression of target organ IFNγ and TNFa mRNA^26^. Therefore, we speculated that HGF secreted by UC-MSCs might be another cytokine responsible for the inhibitory effect of UC-MSCs. Unexpectedly, the addition of HGF hardly altered the expression of functional molecules. Surprisingly, the addition of HGF resulted in a significant increase in the expression of IL10 in monocytes. Consistently, the addition of HGF-blocking antibodies did not alter the expression of functional molecules but significantly downregulated IL10 expression. These data indicate that UC-MSC-derived HGF can induce monocytes to produce IL10, which is in accordance with a previous report on monocytes differentiated in the presence of HGF^27^,^28^.

Several studies have demonstrated that the regulatory properties of MSCs are mediated by IL10 because using antibodies to neutralize IL10 in cocultures of MSCs and immune cell types impaired the immunomodulatory effect of the MSCs. However, these reports were not designed to identify the source of IL10. We found that UC-MSCs did not produce IL10, but that IL10 was exclusively produced by monocytes after exposure to UC-MSCs in our system. The addition of IL10 could block the differentiation of monocytes into DCs, but the inhibition of monocyte differentiation by UC-MSCs was not impaired by using IL10-blocking antibodies. Thus, such increased IL10 production in monocytes after co-culturing with UC-MSCs could further enhance the immunomodulatory effect of UC-MSCs.

In summary, our studies show that UC-MSCs play a powerful suppressive role in multiple immune responses though instructing the generation of IL10-producing, monocyte-derived cells. These results suggest that UC-MSCs may modulate the immune system, not only through acting directly on T cells but also at the first step of the immune response through the inhibition of DC differentiation and maturation, which may help to explain their clinical benefits. Therefore, UC-MSCs may act as powerful regulators of the immune response though triggering a suppressive microenvironment.

## Additional Information

**How to cite this article**: Deng, Y. *et al*. Umbilical Cord-derived Mesenchymal Stem Cells Instruct Monocytes Towards an IL10-producing Phenotype by Secreting IL6 and HGF. *Sci. Rep.* **6**, 37566; doi: 10.1038/srep37566 (2016).

**Publisher's note:** Springer Nature remains neutral with regard to jurisdictional claims in published maps and institutional affiliations.
